# Male Sex Workers Who Sell Sex to Men Also Engage in Anal Intercourse with Women: Evidence from Mombasa, Kenya

**DOI:** 10.1371/journal.pone.0052547

**Published:** 2013-01-02

**Authors:** Priya Mannava, Scott Geibel, Nzioki King’ola, Marleen Temmerman, Stanley Luchters

**Affiliations:** 1 Centre for International Health, Burnet Institute, Melbourne, Victoria, Australia; 2 Population Council, Nairobi, Kenya; 3 International Centre for Reproductive Health (ICRH), Mombasa, Kenya; 4 Department of Obstetrics and Gynaecology, International Centre for Reproductive Health (ICRH), Ghent University, Ghent, Belgium; 5 School of Public Health and Preventive Medicine, Monash University, Victoria, Australia; 6 School of Public Health, University of the Witwatersrand, Johannesburg, South Africa; University of Ottawa, Canada

## Abstract

**Objective:**

To investigate self-report of heterosexual anal intercourse among male sex workers who sell sex to men, and to identify the socio-demographic characteristics associated with practice of the behavior.

**Design:**

Two cross-sectional surveys of male sex workers who sell sex to men in Mombasa, Kenya.

**Methods:**

Male sex workers selling sex to men were invited to participate in surveys undertaken in 2006 and 2008. A structured questionnaire administered by trained interviewers was used to collect information on socio-demographic characteristics, sexual behaviors, HIV and STI knowledge, and health service usage. Data were analyzed through descriptive and inferential statistics. Bivariate logistic regression, after controlling for year of survey, was used to identify socio-demographic characteristics associated with heterosexual anal intercourse.

**Results:**

From a sample of 867 male sex workers, 297 men had sex with a woman during the previous 30 days – of whom 45% did so with a female client and 86% with a non-paying female partner. Within these groups, 66% and 43% of male sex workers had anal intercourse with a female client and non-paying partner respectively. Factors associated with reporting recent heterosexual anal intercourse in bivariate logistic regression after controlling for year of survey participation were being Muslim, ever or currently married, living with wife only, living with a female partner only, living with more than one sexual partner, self-identifying as basha/king/bisexual, having one’s own children, and lower education.

**Conclusions:**

We found unexpectedly high levels of self-reported anal sex with women by male sex workers, including selling sex to female clients as well as with their own partners. Further investigation among women in Mombasa is needed to understand heterosexual anal sex practices, and how HIV programming may respond.

## Introduction

It is now well acknowledged that anal intercourse (AI) is an efficient mode of HIV transmission [1]–[6]. Despite this, the discourse around AI and HIV has largely focused on men who have sex with men, with relatively little evidence available in relation to heterosexual populations. Recent studies have estimated the probability of HIV-1 transmission per unprotected receptive male-to-male AI act at 1.4% [7] and 1.7% per male-to-female AI act [8], compared to a much lower probability of transmission in the range of 0.08–0.3% for receptive vaginal intercourse [8]. These findings suggest that the role of AI in shaping heterosexual HIV epidemics should not be underestimated.

In sub-Saharan Africa, prevention and control of heterosexual HIV transmission has solely revolved around penile - vaginal intercourse despite the evidence of the practice of heterosexual AI [9]. Within various populations sampled in studies undertaken in South Africa, percentages reporting heterosexual AI over different time periods ranged from 43% among female sex workers [10], 42% among truck drivers [11], 10% and 14% among patients of sexually transmitted infection (STI) clinics [12], and 3.6%–15% within the general population [13], [14]. A study on female college students in Togo found that 12% had ever engaged in AI [15], whilst 35% of males and females aged 18–27 years sampled in a large, national Zimbabwean study reported heterosexual AI over the past two months [16]. These findings, coupled with the estimated increased risk of HIV transmission from AI, highlight the need to better understand heterosexual AI in sub-Saharan Africa [17].

In Kenya, little research has been undertaken on heterosexual AI. In two studies, 15% [18] and 41% [19] of female commercial sex workers sampled reported lifetime AI, whilst another study had 4.3% of female sex workers reporting AI during the previous month [20]. Here, we aim to add to the current body of evidence on heterosexual AI in Kenya, and in sub-Saharan Africa overall, from surveys undertaken among male sex workers who sell sex to men in Mombasa, Kenya. These male sex workers are among a subgroup of men who have sex with men with many vulnerabilities and a high prevalence of HIV and other STIs [21]–[23]. A qualitative study exploring the social and behavioral determinants of sexual risks among men who sell sex to men in Mombasa found that male-to-male sex is driven by socio-economic needs, same-sex sexual attraction, or a combination of both [24]. Due to factors ranging from sexual pleasure to maintaining trust in a stable relationship, the majority of men interviewed also reported low levels of consistent condom use with clients as well as regular partners. In addition, stigma and discrimination towards same sex relationships meant that many of the men selling sex to men also maintained heterosexual relationships.

Whilst high levels of unprotected AI with men have been documented among male sex workers in Kenya [25], little is known about their heterosexual behaviors in general and even less about AI with women. There is a need to gain a better understanding of the latter given that sexual networks of men who have sex with men in Kenya also include women from the general population [26].

The objectives of this study were to determine the prevalence of heterosexual AI among male sex workers in Mombasa, and to identify socio-demographic characteristics associated with practice of the behavior.

## Methods

We analyzed data from two surveys undertaken among male sex workers who sell sex to men in Mombasa. These surveys were carried out as part of a larger study evaluating the impact of peer outreach on HIV knowledge and behaviors among male sex workers, the detailed methodology of which is described elsewhere [21].

In brief, the surveys were carried out from October to December 2006 and February to April 2008. Participants were defined as men aged 16 years of older who had recently sold and were currently willing to sell sex to other men in exchange for money and/or goods. These male sex workers were recruited by trained peer mobilizers at known community sites which had been mapped in a previous study [22]: bars, nightclubs and discos, beach areas and beach bars, public streets, public parks, and other private brothels, businesses and estates. Sampling was based on a probability proportional to size approach from a time-venue sampling frame [25]. Peer mobilizers identified the male sex workers based on an initial visual characterization, followed by a casual conversation to confirm that the subject had recent history of and was currently willing to sell sex to men.

Male sex workers who agreed to participate were interviewed by trained interviewers at secure and centrally located venues. Information on participants’ socio-demographic characteristics, self-described sexual identity, sexual behaviors with men and women, HIV prevention knowledge and practices, reported STI symptoms, and exposure to health services was collected based on a structured behavioral questionnaire using handheld computers.

### Ethics Statement

Ethical approval was obtained from the Kenyatta National Hospital Ethics and Review Committee and the Population Council’s Institutional Review Board.

### Statistical Analysis

For the purposes of this study, data on study participants’ socio-economic characteristics and sexual behaviors with women were analyzed. Statistical analysis was carried out using the Intercooled Stata 12.0 statistical package (StataCorp, College Station, Texas). To begin with, the socio-demographic characteristics and heterosexual behaviors of male sex workers were analyzed using descriptive statistics. As described previously [25], here, sexual identity was grouped into two categories: *“Basha, king or bisexual”* who are more likely than other identities to report being insertive partners during anal sex, and *“shoga, queen, gay, homosexual or others”* who are more likely to report being receptive anal sex partners.

Even though the 2006 and 2008 surveys were independent, some male sex workers may have participated in both surveys as personal identifiers were not used. Thus, with the goal of combining both surveys for analysis, we first assessed socio-demographic characteristics and heterosexual behaviors for each survey separately, and compared differences in results using chi-squared tests for categorical variables and Wilcoxon rank-sum test for medians in order to determine whether data from both surveys could be combined in analysis. Following this, the same descriptive analysis was carried out on the entire sample (surveys combined).

In a second analysis which included all participants from both surveys, socio-demographic characteristics associated with recent (during the previous 30 days) vaginal or oral sex, and with recent AI were identified in bivariate logistic regression. The group ‘recent vaginal or oral sex’ was defined as those who reported either or both of those sexual behaviors. This definition did not exclude men who may have also recently practiced AI in addition to vaginal or oral sex. As men who participated in the 2006 survey may have also participated in the 2008 survey, bivariate logistic regression was carried out after controlling for year of survey participation.

## Results

Separate analyses of data from both surveys showed that several socio-demographic characteristics of men surveyed in 2006 were significantly different (p<0.05) from those surveyed in 2008, but that reported sexual behaviors with women were similar (Table 1). This suggested that the data from both samples could be combined. A total of 425 male sex workers were surveyed in 2006, and 442 in 2008.

**Table 1:** Socio-demographic characteristics and reported bisexual behaviours of male sex workers who sell sex to men in Mombasa.

Variable	2006 Survey (n = 425),% (N/D#) or Median(IQR#)	2008 Survey (n = 442),% (N/D#) or Median(IQR#)	Surveys combined (n = 867),% (N/D#) or Median(IQR#)
Median age (years)	26 (22–31)	23 (21–27)*	25 (22–28)
Education level			
None or incomplete primary	38.1 (162/425)	30.3 (134/442)*	34.1 (296/867)
Completed primary or incomplete secondary	40.5 (172/425)	48.9 (216/442)*	44.8 (388/867)
Completed secondary or higher	21.4 (91/425)	20.8 (92/442)*	21.1 (183/867)
Religion			
Muslim	49.5 (204/412)	56.2 (232/413)	52.8 (436/825)
Catholic	30.6 (126/412)	27.1 (112/413)	28.9 (238/825)
Protestant	19.9 (82/412)	16.7 (69/413)	18.3 (151/825)
Sexual self-identification			
Basha/king/bisexual	58.4 (248/425)	56.1 (248/442)	57.2 (496/867)
Shoga/queen/gay/homosexual/others	41.6 (177/425)	43.9 (194/442)	42.8 (371/867)
Ever had sex with a woman	69.2 (285/412)	79.9 (353/442)*	73.6 (638/867)
Ever married to a woman	17.2 (73/425)	22.6 (100/442)*	20.0 (173/867)
Currently married	28.8 (21/73)	36.0 (36/100)	32.9 (57/173)
Currently living with:			
Wife only	3.3 (14/425)	4.5 (20/442)*	3.9 (34/867)
Other female sexual partner only	6.1 (26/425)	8.4 (37/442)*	7.3 (63/867)
Male sexual partner only	22.6 (96/425)	15.6 (69/442)*	19.0 (165/867)
Two or more of the above	2.6 (11/425)	1.1 (5/442)*	1.8 (16/867)
Has children of his own	24.5 (104/425)	26.5 (117/442)	25.5 (221/867)
**Sexual behaviours with female paying clients**			
Reported any sex with a female client in the past 30 days	49.6 (62/125)	41.9 (72/172)	45.1 (134/297)
Median number of female clients in the past 7 days	1 (0–2)	1 (0–2)	1 (0–2)
Reported vaginal intercourse with last female client	82.3 (51/62)	81.9 (59/72)	82.1 (110/134)
Used a condom during vaginal intercourse	68.6 (35/51)	76.3 (45/59)	72.7 (80/110)
Reported AI with last female client	64.5 (40/62)	66.7 (48/72)	65.7 (88/134)
Used a condom during AI	60.0 (24/40)	60.4 (29/48)	60.2 (53/88)
Last female client was Kenyan	88.7 (55/62)	88.9 (64/72)	88.8 (119/134)
Reported any sex with a female non-paying partner in the past 30 days	86.4 (108/125)	85.5 (147/172)	85.9 (255/297)
Median number of female non-paying partners in the past 7 days	1 (0–2)	1 (0–1)	1 (0–1)
Reported vaginal intercourse with last female non-paying partner	98.1 (101/103)	97.9 (139/142)	98.0 (240/245)
Used a condom during vaginal intercourse	65.3 (66/101)	55.4 (77/139)	59.6 (143/240)
Reported AI with last female non-paying partner	40.6 (43/106)	44.5 (65/146)	42.9 (108/252)
Used a condom during AI	55.8 (24/43)	52.3 (34/65)	53.7 (58/108)
Last female non-paying partner was Kenyan	96.3 (104/108)	93.2 (137/147)	94.5 (241/255)

# N = numerator, D = denominator, IQR = inter-quartile range.

* Variables for which the P-value was statistically significant (*P*<0.05) when testing for differences between values obtained in the 2006 and 2008 surveys. Wilcoxon rank-sum test used for median values, chi-squared tests for categorical variables.

The median age of participants was 25 years (IQR = 22–28), and nearly half (45%) had completed primary education (Table 1). Just over half (53%) were Muslim and self-identified themselves as basha, king, or bisexual (57%). Close to 75% of participants reported ever having had sex with a woman, and a much smaller percentage, 20%, were ever married to a woman. Nineteen percent of the entire sample were living with a male sexual partner only, and 7% with a female sexual partner only. Close to 2% were living with more than one sexual partner, or with a sexual partner and their wife. One quarter of male sex workers stated that they had children of their own.

A third of male sex workers (34%, n = 297) within the combined sample recently had sex with a woman. More specifically, within this group of men, 45% had recent sex with a female client and 86% with a non-paying female partner. Thirty-one percent had sex with both a female client and a non-paying partner. Vaginal intercourse was widely practiced, with over 80% of men in both groups (female client or non-paying partner) reporting this behavior. Significant percentages of men also reported having heterosexual AI: 66% (88/134) of those who recently had sex with a female client, and 43% (108/252) of those who recently had sex with a non-paying female partner. In the former group, 60% of men used a condom during AI, whilst in the latter group the figure was 54%. Reported condom use was higher for vaginal intercourse, at 73% for men who had sex with female clients and 60% for those who had sex with non-paying female partners.

Table 2 shows the socio-demographic characteristics associated with report of recent AI in male sex workers who sell sex to men, after controlling for survey year in bivariate logistic regression. Characteristics associated with AI were being currently married (OR, 2.72; 95% CI, 1.24–5.98), having ever been married (OR, 3.01; 95% CI, 1.99–4.54), and having children of their own (OR, 3; 95% CI, 2.02–4.46). In addition, men who reported recent AI were more likely to be Muslim than Protestant (OR, 4.10; 95% CI, 2.12–7.93), self-identify as basha/king/bisexual rather than shoga/queen/gay/homosexual (OR, 4.63; 95% CI, 3.03–7.06), be living with their wife only, a female sexual partner only, or a combination of sexual partners rather than with a male sexual partner only (OR, 10.23; 95% CI, 3.80–27.56; OR, 14.48; 95% CI, 6.25–33.54; OR, 16.56; 95% CI, 4.47–61.29 respectively), and less likely to have completed secondary or higher education than primary education (OR, 0.60; 95% CI, 0.37–1.00).

**Table 2:** Recent (past 30 days) vaginal and/or oral sex and AI outcomes by selected socio-demographic characteristics in bivariate logistic regression after controlling for survey year, among male sex workers who sell sex to men in Mombasa.

Variable	Recent vaginal and/or oral sex (n = 285),% (N/D#) or Median(IQR#)	OR† (95% CI)	Recent AI (n = 156),% (N/D#) or Median(IQR#)	OR† (95% CI)
Median age (years)	25 (22–29)	1.25**°** (0.94–1.68)	25 (21–29.5)	1.29**°** (0.90–1.86)
Education level				
None or incomplete primary	32.4 (95/293)	0.86 (0.62–1.18)	20.8 (52/250)	0.84 (0.56–1.25)
Completed primary or incomplete secondary	36.8 (141/383)	(Ref)	24.6 (79/321)	(Ref)
Completed secondary or higher	27.4 (49/179)	0.66* (0.45–0.97)	16.1 (25/155)	0.60* (0.37–1.00)
Religion				
Muslim	38.5 (165/429)	2.05*** (1.33–3.16)	28.6 (106/370)	4.10*** (2.12–7.93)
Catholic	28.4 (67/236)	1.33 (0.82–2.14)	15.5 (31/200)	1.90 (0.92–3.95)
Protestant	23.0 (34/148)	(Ref)	8.8 (11/125)	(Ref)
Sexual self-identification				
Basha/king/bisexual	46.5 (226/486)	4.70*** (3.37–6.56)	32.1 (123/383)	4.63*** (3.03–7.06)
Shoga/queen/gay/homosexual/others	16.0 (59/369)	(Ref)	9.6 (33/343)	(Ref)
Ever married to a woman	54.1 (93/172)	2.94*** (2.08–4.15)	39.7 (52/131)	3.01*** (1.99–4.54)
Currently married	71.4 (40/56)	3.10*** (1.55–6.21)	56.8 (21/37)	2.72* (1.24–5.98)
Currently living with:				
Wife only	69.7 (23/33)	10.86*** (4.63–25.46)	54.6 (12/22)	10.23*** (3.80–27.56)
Other female sexual partner only	75.0 (45/60)	14.18*** (6.91–29.11)	62.5 (25/40)	14.48*** (6.25–33.54)
Male sexual partner only	17.8 (29/163)	(Ref)	10.7 (16/150)	(Ref)
Two or more of the above	75.0 (12/16)	13.71*** (4.12–45.61)	66.7 (8/12)	16.56*** (4.47–61.29)
Has children of his own	54.3 (119/219)	2.93*** (2.13–4.04)	38.7 (63/163)	3.00*** (2.02–4.46)

# N = numerator, D = denominator, IQR = inter-quartile range.

† Odds ratios calculated after controlling for year of survey in bivariate logistic regression.

°Ratio measures odds of recent vaginal/oral sex or recent AI for those whose age is above the median of 25 years.

* *P*<0.05,

** *P*<0.01,

*** *P*<0.001.

## Discussion

This study is one of the first to examine heterosexual AI behaviors among male sex workers who sell sex to men in Kenya. Our results show that whilst vaginal intercourse is the more widely practiced heterosexual behavior among male sex workers who sell sex to men in Mombasa, a substantial number of these men are also engaging in AI with women. The proportion of men in this sample reporting heterosexual AI was 65% among men who sold sex to a female client and 43% among men who had sex with a non-paying female partner. We also found that condom use in AI was lower than in vaginal intercourse, which may be due to a general perception that anal sex is safer than vaginal sex as was reported in qualitative discussions with the male sex workers [24]. These results are in line with those from another diary study undertaken among a small cohort of male sex workers in Mombasa, which found that 54% of 215 sexual encounters with women comprised AI. Of these anal sex acts, only 54% were protected – a figure which was lower than that obtained for vaginal sex at 62% [27].

In addition, this study found that, male sex workers engaging in heterosexual AI were more likely to be ever or currently married, living with their wife only, a female sexual partner only, or several sexual partners (male and female) as opposed to with a male sexual partner only, having children of their own, and to self-identify as basha/king/bisexual. These findings suggest that male sex workers who practice bisexual behaviors show closer interactions with women in their daily lives. As mentioned previously, male sex workers in Mombasa who self-identify as basha, king, or bisexual are more likely to be an insertive partner during anal sex with men than other identities [25]. Not surprisingly this analysis also found that bashas/kings/bisexuals were also more likely to report vaginal or anal sex with female partners. These male sex workers likely play a more masculine sexual role with both male and female partners, but more qualitative inquiry is needed to understand these complex social, sexual, and commercial interactions and their public health implications. Additional factors associated with heterosexual AI were being Muslim rather than Protestant, and decreased likelihood of having completed secondary or higher education compared to primary education. There is no evidence to explain why Muslim respondents were more likely to engage in heterosexual AI, although key informants anecdotally report that some young Muslim women in Coastal Kenya may be using AI as a “virginity preservation” strategy to avoid vaginal intercourse. More qualitative enquiry into this possible explanation is needed.

Given that men who have sex with men are at higher risk of HIV infection than general populations [28], these male sex workers may be acting as a bridge for HIV transmission between higher-risk and lower-risk heterosexual female populations. Paying female clients may also be populations at higher risk of HIV than non-paying female partners. More detailed study of sexual networking among male sex workers, their male and female clients, and non-paying partners are needed to understand the dynamics of HIV transmission among these groups.

The surveys were based on self-reported data of sexual behaviors that are stigmatized, and thus subject to underreporting. Similarly, recall bias may have also resulted in an overestimation or underestimation of the frequencies reported here. Another limitation of this study was the inability to compare socio-demographic characteristics of male sex workers who only had anal sex with those who only had vaginal or oral sex, due to small sample sizes. Lastly, whilst we attempted to address repeat participation in the surveys by controlling for year of the survey in logistic regression analyses, it must still be noted that combining both surveys may have also resulted in overestimation or underestimation of frequencies.

To conclude, we found that male sex workers who sell sex to men in Mombasa are also engaging in sex with women, including AI. The reported low condom use in AI, coupled with the high rate of HIV transmission associated with the behavior, suggest that a potential route of HIV transmission is being overlooked. Thus, there may be an under-assessment of heterosexual AI behaviors among different populations, requiring more research to inform HIV prevention programming. In particular, data on the characteristics of women engaging in or paying male sex workers for AI was not collected in this study, and thus there is a need to gain a better understanding of these women.

## References

[1] Winklestein W Jr, Lyman DM, Padian N, Grant R, Samuel M, et al (1987) Sexual practices and risk of infection by the human immunodeficiency virus: The San Francisco Men’s Health Study. JAMA 257: 321–325. 3540327

[2] Lazzarin A, Saracco A, Musicco M, Nicolosi A (1991) Man-to-woman sexual transmission of the human immunodeficiency virus: Risk factors related to sexual behavior, man’s infectiousness, and women’s susceptibility. Arch. Intern. Med 151: 2411–2416. 1684098

[3] Darrow WW, Echenberg DF, Jaffe HW, O’Malley PM, Byers RH, et al (1987) Risk factors for human immunodeficiency virus infections in homosexual men. Am J Public Health 77: 479–483. 3030146 10.2105/ajph.77.4.479 PMC1646936

[4] Seidlin M, Vogler N, Lee E, Lee YS, Dubin N (1993) Heterosexual transmission of HIV in a cohort of couples in New York City. AIDS 7: 1247–1254. 8216983 10.1097/00002030-199309000-00015

[5] European Study Group on Heterosexual Transmission of HIV (1992) Comparison of female to male and male to female transmission of HIV in 563 stable couples. BMJ 304: 809–813. 1392708 10.1136/bmj.304.6830.809 PMC1881672

[6] Padian N, Marquis L, Francis DP, Anderson RE, Rutherford GW, et al (1987) Male-to-female transmission of human immunodeficiency virus. JAMA 258: 788–790. 3475478

[7] Baggaley RF, White RG, Boily MC (2010) HIV transmission risk through anal intercourse: systematic review, meta-analysis and implications for HIV transmission. Int J Epidemiol 39(4): 1048–63. 20406794 10.1093/ije/dyq057 PMC2929353

[8] Boily MC, Baggaley RF, Wang L, Masse B, White RG, et al (2009) Heterosexual risk of HIV-1 infection per sexual act: systematic review and meta-analysis of observational studies. Lancet Infect Dis 9: 118–29. 19179227 10.1016/S1473-3099(09)70021-0 PMC4467783

[9] Brody S, Potterat JJ (2003) Assessing the role of anal intercourse in the epidemiology of AIDS in Africa. Int J STD & AIDS 14: 431–436. 12869220 10.1258/095646203322025704

[10] Morar NS, Ramjee G, Abdool Karim SS (1998) Vaginal insertion and douching practices among sex workers at truck stops in KwaZulu-Natal. S Afr Med J 88(4): 470. 9594997

[11] Ramjee G, Gouws E (2002) Prevalence of HIV among truck drivers visiting sex workers in KwaZulu-Natal, South Africa. Sex Transm Dis 29: 44–9. 11773878 10.1097/00007435-200201000-00008

[12] Kalichman SC, Simbayi LC, Cain D, Jooste S (2009) Heterosexual anal intercourse among community and clinical settings in Cape Town, South Africa. Sex Transm Infect 85: 411–5. 19429569 10.1136/sti.2008.035287 PMC3017216

[13] Kalichman SC, Pinkerton SD, Carey MP, Cain D, Mehlomakulu V, et al (2011) Heterosexual anal intercourse and HIV infection risks in the context of alcohol serving venues, Cape Town, South Africa. BMC Public Health 11: 807. 21999574 10.1186/1471-2458-11-807 PMC3207968

[14] Lane T, Pettifor A, Pascoe S, Fiamma A, Rees H (2006) Heterosexual anal intercourse increases risk of HIV infection among young South African men. AIDS 20(1): 123–5. 16327330 10.1097/01.aids.0000198083.55078.02

[15] Sallah ED, Grunitzky-Bekele M, Bassabi K, Dodzro K, Sadzo A, et al.. (1999) Sexual behavior, knowledge and attitudes to AIDS and sexually transmitted diseases of students at the University of Benin (Togo). Sante 9: 101–9 [cited in Brody & Potterat 2003]. 10377497

[16] Akande A, Muvakure G, Nyanhete P, Williams B, Chitumba B, et al (1993) AIDS high-risk and precautionary behaviour in young African adults: a Zimbabwean perspective. Early Child Dev Care 88: 73–85.

[17] Boily MC, Baggaley RF, Masse B (2009) The role of heterosexual anal intercourse for HIV transmission in developing countries: are we ready to draw conclusions? Sex Transm Infect 85(6): 408–10. 19826062 10.1136/sti.2009.037499 PMC3395196

[18] Fonck K, Kaul R, Keli F, Bwayo JJ, Ngugi EN, et al (2001) Sexually transmitted infections and vaginal douching in a population of female sex workers in Nairobi, Kenya. Sex Transm Infect 77(4): 271–5. 11463927 10.1136/sti.77.4.271 PMC1744330

[19] Schwandt M, Morris C, Ferguson A, Ngugi E, Moses S (2006) Anal and dry sex in commercial sex work, and relation to risk for sexually transmitted infections and HIV in Meru, Kenya. Sex Transm Infect 2(5): 392–6. 10.1136/sti.2006.019794 PMC2563859 16790563

[20] Veldhuijzen NJ, Ingabire C, Luchters S, Bosire W, Braunstein S, et al (2011) Anal intercourse among female sex workers in East Africa is associated with other high-risk behaviours for HIV. Sex Health 8(2): 251–4. 21592442 10.1071/SH10047

[21] Geibel S, King’ola N, Temmerman M, Luchters S (2012) The impact of peer outreach on HIV knowledge and prevention behaviors of male sex workers in Mombasa, Kenya. Sex Transm Infect DOI: 10.1136/sextrans-2011-050224. 10.1136/sextrans-2011-050224 22332149

[22] Geibel S, van der Elst EM, King’ola N, Luchters S, Davies A, et al (2007) ‘Are you on the market?’: a capture-recapture enumeration of men who sell sex to men in and around Mombasa, Kenya. AIDS 21: 1349–54. 17545712 10.1097/QAD.0b013e328017f843

[23] Luchters S, Geibel S, Syengo M, Lang’o D, King’ola N, et al (2011) Use of AUDIT, and measures of drinking frequency and patterns to detect associations between alcohol and sexual behavior in male sex workers in Mombasa, Kenya. BMC Public Health 11(1): 384. 21609499 10.1186/1471-2458-11-384 PMC3128017

[24] Okal J, Luchters S, Geibel S, Chersich MF, Lango D, et al (2009) Social context, sexual risk perceptions and stigma: HIV vulnerability among male sex workers in Mombasa, Kenya. Culture, Health and Sexuality 11(8): 811–26. 10.1080/13691050902906488 19484638

[25] Geibel S, Luchters S, King’ola N, Esu-Williams E, Rinyiru A, et al (2008) Factors associated with unprotected anal sex among male sex workers in Mombasa, Kenya. Sex Transm Dis 35(8): 746–52. 18650772 10.1097/OLQ.0b013e318170589d

[26] Onyango-Ouma W, Birungi H, Geibel S (2005) Understanding the HIV/STI risks and prevention needs of men who have sex with men in Nairobi, Kenya. Washington: Population Council.

[27] Smith A, Muhaari A, Agwanda C, Kowuor D, van der Elst E, et al. (2010) Female clients and partners of MSM sex workers in Mombasa, Kenya [Abstract]. 17^th^ Conference on Retroviruses and Opportunistic Infections, San Francisco, CA; February 12, 2010. [Paper # 39].

[28] Beyrer C, Baral SD, van Griensven F, Goodreau SM, Chariyalertsak S, et al (2012) Global epidemiology of HIV infection in men who have sex with men. The Lancet 380(9839): 367–77. 10.1016/S0140-6736(12)60821-6 PMC3805037 22819660

